# Can counter-advertising diminish persuasive effects of conventional and pseudo-healthy unhealthy food product advertising on parents?: an experimental study

**DOI:** 10.1186/s12889-020-09881-1

**Published:** 2020-11-25

**Authors:** Helen Dixon, Maree Scully, Claudia Gascoyne, Melanie Wakefield

**Affiliations:** 1 Centre for Behavioural Research in Cancer, Cancer Council Victoria, 615 St Kilda Road, Melbourne, Victoria 3004 Australia; 2 Melbourne School of Psychological Sciences, The University of Melbourne, Parkville, Victoria 3010 Australia

**Keywords:** Food advertising, Counter-advertising, Parents, Experiment, Unhealthy food, Energy-dense nutrient-poor food

## Abstract

**Background:**

To help address rising rates of obesity in children, evidence is needed concerning impacts of common forms of marketing for unhealthy child-oriented food products and the efficacy of educational interventions in counteracting any detrimental impacts of such marketing. This study aims to explore parents’ responses to advertising for unhealthy children’s food products that employ different types of persuasive appeals and test whether a counter-advertising intervention exposing industry motives and marketing strategies can bolster parents’ resistance to influence by unhealthy product advertising.

**Methods:**

*N* = 1613 Australian parents were randomly assigned to view online either a: (A) non-food ad (control); (B) conventional confectionery ad (highlighting sensory benefits of the product); (C) pseudo-healthy confectionery ad (promoting sensory benefits and health attributes of the product); (D) conventional confectionery ad + counter-ad (employing inoculation-style messaging and narrative communication elements); (E) pseudo-healthy confectionery ad + counter-ad. Parents then viewed various snacks, including those promoted in the food ads and counter-ad. Parents nominated their preferred product, then rated the products.

**Results:**

Exposure to the conventional confectionery ad increased parents’ preference for the advertised product, enhanced perceptions of the product’s healthiness and reduced sugar content and boosted brand attitude. Exposure to the pseudo-healthy confectionery ad increased parents’ preference for the advertised product, and enhanced perceptions of healthiness, fibre content and lower sugar content. The counter-ad diminished, but did not eliminate, product ad effects on parents’ purchasing preference, product perceptions and brand attitudes. The counter-ad also prompted parents to perceive processed foods as less healthy, higher in sugar and lower in fibre and may have increased support for advertising regulation.

**Conclusions:**

Exposure to unhealthy product advertising promoted favourable perceptions of products and increased preferences for advertised products among parents. Counter-advertising interventions may bolster parents’ resistance to persuasion by unhealthy product advertising and empower parents to more accurately evaluate advertised food products.

**Supplementary Information:**

The online version contains supplementary material available at 10.1186/s12889-020-09881-1.

## Introduction

Commercial marketing of children’s food products has been scrutinised as a potential contributor to the childhood obesity epidemic for its role in promoting excess consumption of energy-dense, nutrient-poor (unhealthy) foods. To help address rising rates of obesity in children, evidence is needed concerning impacts of common forms of marketing for unhealthy child-oriented foods and the efficacy of interventions in counteracting any detrimental impacts of such marketing. The present study assesses parents’ reactions to unhealthy food advertisements (ads) and whether counter-advertising strategies can bolster their resistance to being influenced by the former.

Food marketing communications are typically used to build brand awareness and enhance consumers’ expectations of the sensory (e.g. flavour, texture) and non-sensory benefits (e.g. social and symbolic value) of purchasing and consuming a given product [1–3]. Numerous studies have found the most heavily advertised food products are those we should avoid (i.e. energy-dense, nutrient-poor foods) while healthy, whole foods are under-represented in the food marketing landscape [4–7]. In terms of marketing of children’s food products, there appears to have been a shift over recent years in some of the persuasive appeals used [8, 9]. While advertising for these products continues to emphasise sensory and social enjoyment of advertised products [1, 10], it is now common for such advertising to highlight health and nutritional product attributes as well – even if the product is energy-dense and nutrient-poor [11–14]. Many of the latter type of persuasive appeals appear to be directed at parents [15]. The present study is concerned with investigating parents’ reactions to these different types of persuasive appeals commonly used in television advertising for children’s food products.

Much research and debate about potential detrimental impacts of food marketing has focused on children, with a recent systematic review finding that marketing for unhealthy foods enhances attitudes and preferences, and increases consumption of advertised foods [10]. Children may be especially vulnerable to persuasion by food advertising because their immature cognitive abilities mean they may be unaware of the persuasive intent of advertising, plus they may be especially responsive to the types of persuasive appeals commonly employed in food advertising [2, 16]. However, to comprehensively assess how food marketing may affect children’s diets, it is also important to consider how food marketing affects parents, as they control most household food purchasing and preparation and are important role models and gate keepers for their children’s diets [17–20]. This parental role, coupled with growing public opposition to child-targeted food marketing, means parents are an increasingly important target group for food marketing [21]. While parents would be more aware of the persuasive intent of food marketing than children, they are not immune to its influence [22–24].

Health-oriented persuasive appeals used to market unhealthy products may lead parents to erroneously believe such products to be healthy. Previous research has found that branding and labelling emphasising one aspect of a food product as healthy can create a ‘health halo’, leading people to generalise that the food is favourable on a range of nutritional attributes [25]. In Australia, claims that a high-sugar product made from concentrated fruit paste was ‘made with 65% real fruit’ were deemed potentially misleading and deceptive by the Australian Competition and Consumer Commission in 2005 and the company responsible was required to modify its advertising and marketing practices to be more accurate [26]. In the present study, we test how parents respond to such claims and other common persuasive appeals used to advertise child-oriented food products.

Given the high frequency, reach and detrimental impact of unhealthy food product advertising on people’s diets [6, 7, 27, 28], population-level approaches will be required to effectively eliminate, modify or counter its influence. Various methods of training, intervention and regulation may help people to resist unwanted persuasion from advertising exposure and assist them to carry out more deliberate, informed decision-making [29]. Regulations banning or restricting certain forms of food advertising are one potential method for eliminating or reducing the public’s exposure to unhealthy food advertising [5]. Such regulations have been implemented in some jurisdictions [30], with evidence from Canada indicating they can effectively reduce children’s exposure to unhealthy food advertising [31]. However, overall there has been variable progress achieved globally in restricting the marketing of high-fat, sugary and salty food and beverage products to children [32].

In the absence of policy to restrict the amount or type of food marketing, an alternative path is to provide consumers with information and skills designed to empower them to be less susceptible to influence by unhealthy food marketing and more accurately evaluate the nutritional value of food products. Product labelling such as nutrition information panels, front-of-pack nutrition labelling, or warning labels are important sources of information that may assist consumers in more accurately evaluating food products [33]. However, in contexts where unhealthy food marketing is prolific, consumers may benefit from additional information and guidance to help them evaluate advertised products. Media literacy education offers one such approach, by seeking to build awareness of media influence and encouraging people to actively and critically consume media. Media literacy interventions (typically delivered to children via the school curriculum) have been used to bolster resistance to marketing, with meta-analyses and reviews indicating small, positive impacts [29, 34]. In a recent experimental trial, adults were exposed to a media literacy intervention about sugar-sweetened beverage advertising via a series of small group education sessions, teach back calls and interactive phone calls. The intervention enhanced their ability to critically evaluate sugar-sweetened beverage advertisers’ motives and messaging, and detect missing health information, irrespective of their baseline level of health literacy [35]. While media literacy education appears to be helpful in empowering consumers to more critically evaluate food marketing, it tends to be delivered in a fairly resource intensive manner (i.e. face to face groups run over a number of sessions). Additional approaches that can be delivered én masse (e.g. via mass media) could potentially have broader population reach and impact.

Counter-advertising is one such potential technique, whereby advertising and health communication strategies are used to reduce demand for unhealthy products by exposing the motives and marketing activities of their producers [36]. In previous research, we found that parents shown online counter-advertisements debunking potentially misleading marketing claims were less influenced by front-of-pack promotions on unhealthy foods and more accurately evaluated how nutritious the promoted food products were than parents who did not see such counter-advertising [37]. However, this strategy showed mixed efficacy with pre-adolescent children, since only children who understood the counter-ads responded as intended, whereas children who misunderstood the counter-ads were not protected from the influence of the front-of-pack promotions on unhealthy foods [38].

Another theoretically-based approach that may be used to instil resistance to unwanted persuasion involves use of inoculation messages [39]. This approach draws on principles of social influence, persuasion and message processing and has been applied to various domains, including politics, cross-cultural relations and health risk communication on tobacco, alcohol, safer sex and vaccination [40]. Inoculation messages usually comprise a threat (making people aware that their view on an issue is vulnerable to persuasive attacks) and a refutational pre-emption (introducing and refuting counterarguments against one’s position) [41]. Meta-analyses have found inoculation to be an effective strategy to induce resistance to persuasion [29, 42]. Application of inoculation messaging to bolstering consumer resistance to unhealthy food marketing is somewhat novel. Mason & Miller found print-based inoculation messages could confer resistance to potentially deceptive health and nutrition related claims used in commercial food advertising among a sample of 167 adult undergraduate students [43].

Another perspective that could inform strategies for empowering consumers to be less vulnerable to influence by marketing for unhealthy food is ‘anti-consumerism’. This socio-political position argues that business corporations pursue financial and economic advantage to the detriment of the health and welfare of society, the environment and animals [44]. Anti-consumerists criticise advertising for its use of unrealistic, escapist messages that exploit people’s insecurities by implying that owning advertised products will enhance a person’s image, popularity and happiness [45]. Anti-consumerist social movements often use a process known as ‘culture jamming’ to criticise and subvert mass media advertising and consumerism, using methods such as creating advertising that parodies global brands as a form of media activism (a.k.a. ‘subvertising’) [46, 47]. Applied to food marketing, the anti-consumerist perspective may be used to critically consider the role of the ultra-processed food industry in contributing to the global obesity epidemic (an argument supported by prominent public health experts [48]), and calling into question the marketing tactics and advertising appeals this industry uses to promote their products to consumers. Anti-consumerist movements have given rise to some powerful examples of cultural jamming critiquing major multinational food and beverage brands [49, 50].

Despite differing terminology and disciplinary origins, there is some conceptual overlap in the various approaches outlined above. Each recognises the potential persuasive power of mass media communications and advertising and seeks to deconstruct, expose and contest the values and messages underlying product advertising in order to inform and empower audiences to be less susceptible to advertising influence.

In the present study, we assess whether a counter-ad can empower parents to be less susceptible to influence by advertising for unhealthy child-oriented food products. The counter-ad we test (‘Our kids are sweet enough’) was produced by the Obesity Policy Coalition in Australia for real-world dissemination. While this counter-ad was not developed with a particular theoretical orientation in mind, it employs narrative communication and inoculation-style messaging to expose the potentially misleading marketing practices of the ultra-processed food industry, told through an animated story about a fictional character called ‘Alfie the Apple’. Health communicators often use narrative communication in the form of entertainment education and storytelling centred on the experiences of one or more real or fictional characters to persuade, motivate and support healthy behaviour [51, 52]. Transportation-imagery theory and the extended Elaboration Likelihood Model predict that narrative communications are persuasive because audience identification with featured characters using a familiar mode of human interaction facilitates absorption in the story [51]. A meta-analysis of 25 studies found use of narrative in health communication can impact persuasion, particularly if narratives are delivered via audio and video rather than print [53]. Similarly, Braddock & Dillard’s meta-analysis of 74 studies testing narratives on various communication topics found evidence supportive of a persuasive impact on beliefs, attitudes, intentions and behaviours [54].

Thus the aims of this study are two-fold:

1. Assess parents’ ***responses to product advertising*** for energy-dense, nutrient-poor ‘unhealthy’ children’s food products that employs different types of persuasive appeals:
  *Conventional confectionery:* highlighting sensory benefits such as colour, flavour, shape and fun;
  *Pseudo-healthy confectionery:*promoting sensory benefits and health attributes such as ‘no artificial colours or flavours’ and ‘made with real fruit’.
2. Test the ***efficacy of counter-advertising*** employing inoculation-style messaging and narrative communication elements in bolstering parents’ resistance to being influenced by the above types of product advertising.

We hypothesised that exposure to both types of confectionery advertising (conventional and pseudo-healthy) would promote increased purchasing preferences (H1a) and more favourable brand attitudes (H1b) towards the advertised confectionery products than exposure to control advertising. We further predicted that exposure to pseudo-healthy confectionery advertising would enhance perceptions of the health and nutritional attributes of advertised products (H1c) compared to control advertising. As counter-advertising is intended to bolster consumers’ resistance to being misled by deceptive marketing messages, we hypothesised that exposure to a counter-ad would reduce promotional effects of the product advertising (H2a) and increase purchasing preferences for healthier snack choices (H2b). Finally, we tested whether viewing the counter-ad promoted increased support for policy concerning food labelling and marketing (RQ1).

## Method

### Design and procedure

A between-subjects online survey experimental design was employed whereby parents were randomly assigned to one of five advertising conditions: (A) non-food advertising (control); (B) conventional confectionery advertising; (C) pseudo-healthy confectionery advertising; (D) conventional confectionery + counter-advertising; (E) pseudo-healthy confectionery + counter-advertising. After viewing their assigned ads, parents were shown different types of snacks, including those promoted in the food product advertising (conventional confectionery, pseudo-healthy confectionery) and the counter-advertising (whole fruit) and asked to select which snack they would be most likely to buy for their child. This purchasing preference task was done in two steps: firstly with generic, unbranded products; then with branded products. After each step, parents completed questions assessing their perceptions of the healthiness and nutrition content of each of the snacks shown. Ethical approval for the study was obtained from Cancer Council Victoria’s Institutional Research Review Committee (IER 1810).

### Participants

A sample of Australian parents of children aged 5 to 12 years was recruited through two national, non-probability online panels managed by i-Link Research and Lightspeed. Each panel comprises members who have opted in to receive invitations to participate in research and receive points for completing surveys that can be redeemed for rewards. Deduplication systems were put in place to identify and remove duplicate participants across the two panels. To detect small effect sizes which are typical in experimental media research [55, 56], it was estimated a minimum of *n* = 240 parents per condition would need to be recruited (i.e. *N* ≥ 1200 overall) [57]. We achieved a final sample size of *N* = 1613 (*n* ≥ 320 per condition). A total of 11,853 panellists accessed the survey between 13th August and 6th September 2019. General information about the study was provided to panellists at the start of the survey (i.e. that their participation would involve answering a range of questions about demographics and food products) in order to obtain their informed consent to participate. Panellists who consented to participate (*n* = 10,682) were then screened and designated as ineligible to participate in the study if they were: not the parent/guardian of at least one child aged between 5 and 12 years (*n* = 7224); not the main or joint grocery buyer for their household (*n* = 711); employed (or had close family or friends) in the food manufacturing or marketing industries or dietitians/nutritionists (*n* = 275); or unable to see or hear the video check question (*n* = 471). A further 363 panellists abandoned the survey before completion while 25 were excluded following standard quality control processes, resulting in our final sample of 1613 parents. The demographic profile of the sample is summarised in Table 1.

**Table 1:** Sample characteristics by advertising condition

	Total (*N* = 1613)	Advertising condition				
		Non-food (control) (*n* = 322)	Conventional confectionery (*n* = 323)	Pseudo-healthy confectionery (*n* = 323)	Conventional confectionery + counter-ad (*n* = 324)	Pseudo-healthy confectionery + counter-ad (*n* = 321)
	%	%	%	%	%	%
Sex						
Male	43.5	43.8	43.3	43.3	43.8	43.3
Female	56.4	55.9	56.7	56.7	56.2	56.7
Other	0.1	0.3	0.0	0.0	0.0	0.0
Age group						
< 35 years	31.7	32.3	32.2	31.3	31.2	31.5
35–44 years	41.3	41.0	40.9	41.5	42.0	41.1
45 years or older	27.0	26.7	26.9	27.2	26.9	27.4
Highest level of education						
Secondary school or less	19.0	15.5	20.4	17.3	19.4	22.1
TAFE or Trade Certificate or Diploma	28.2	34.2	25.4	27.2	26.9	27.4
University degree	52.8	50.3	54.2	55.4	53.7	50.5
Socio-economic position (area-based)^a^						
Low (1–33%)	26.9	30.4	26.0	25.1	25.0	28.0
Medium (34–67%)	33.5	31.4	31.3	33.7	38.0	33.3
High (68–100%)	39.6	38.2	42.7	41.2	37.0	38.6
Number of children aged 5–12 years						
One	69.1	72.4	64.7	68.1	70.1	70.4
Two or more	30.9	27.6	35.3	31.9	29.9	29.6

Percentages may not sum to 100% due to rounding

^a^ Socio-economic position was determined according to the Australian Bureau of Statistic’s Index of Relative Socio-Economic Disadvantage ranking for Australia using participant’s residential postcode [58]. This index ranks areas on a continuum of disadvantage (from most disadvantaged to least disadvantaged) taking into consideration characteristics that may enhance or reduce socio-economic conditions of the area

### Advertising stimuli

#### Product advertising

Parents were shown 30 s of existing television advertising for either conventional confectionery (conditions B and D), pseudo-healthy confectionery (conditions C and E) or a department store (condition A). Both types of confectionery advertising featured parents and children, but each used slightly different persuasive strategies to promote the child-oriented food product. The conventional confectionery advertising was a single 30-s ad that highlighted sensory attributes of the product (e.g. colour, flavour and shape) and included themes of fantasy and fun. It featured a giant puppet doll walking through a city (to the tune of a popular nursery rhyme) and showering the crowd of people beneath her with a popular brand of jelly confectionery. Within the crowd the ad zooms in on several parent child dyads enjoying the magical moment together (e.g. a father carrying his son on his shoulders reaches up, catches a sweet and hands it to his son who eats it). The ad concludes with a shot of the confectionery brand logo and the caption, “*<Brand name> makes smiles”.* The pseudo-heathy confectionery advertising consisted of two 15-s ads for a brand of ultra-processed, sugary ‘fruit’ snacks that employed similar persuasive appeals to the conventional confectionery ad (e.g. flavour, fun) with an additional focus on health. These ads depicted a young child at play with friends and responding to their mother’s call that it is time to go by holding up an opened <Brand Name> fruit strap/leather. The mother then happily lets their child continue playing. During both ads, a male voiceover states “<Brand name> have 40% less sugar than other leading kids’ snack products. So all <Brand name> come with a licence to play”. Due to constraints in identifying confectionery advertising that exemplified the two types of persuasive strategies being examined, it was not possible to match the selected confectionery products on characteristics such as advertising expenditure or sales. However, both products and their associated food company brand are well-known in the Australian marketplace. The control non-food advertising consisted of two 15-s ads promoting the <Brand name> Big Brand Toy Sale, showcasing popular items such as gaming consoles, action figures, scooters and building blocks.

#### Counter-advertising

After viewing their assigned product advertising, parents in conditions D and E (counter-advertising intervention) were shown the ‘Our kids are sweet enough’ video produced by the Obesity Policy Coalition in Australia (see https://www.opc.org.au/what-we-do/kids-are-sweet-enough). This video (1 min 45 s in length) aims to shed light on the deceptive marketing tactics food companies employ to promote high-sugar children’s food products to parents. See Additional file 1 for voice-over script and still images from the counter-ad. The ‘Our kids are sweet enough’ counter-ad employs narrative communication to tell the story of ‘Alfie the Apple’ who undergoes food processing that renders him high in sugar and low in fibre, warning viewers of potentially misleading strategies food marketers use to market unhealthy foods. This counter-ad also contains message elements reflective of inoculation messages. Firstly, it raises a threat alerting the viewer that their current beliefs may be vulnerable to persuasive attack. The counter-ad begins by endorsing widely held knowledge that whole fruit and vegetables are nutritious and healthy. It then raises the threat that this assumption is being undermined by the ultra-processed food industry, whose manufacturing processes strip the goodness out of whole foods, and whose misleading marketing strategies promote the idea that the resultant ultra-processed foods are still good for you. The counter-ad then engages in refutational pre-emption by introducing and refuting counter-arguments against conventional wisdom about whole foods being best. Viewers are reminded that healthy foods are more nutritious than energy-dense, nutrient-poor ultra-processed foods, and that despite what industry marketing might claim, you can’t believe it. Viewers are urged to stick to their position and not be swayed by misleading advertising claims.

Parents in conditions A-C (counter-advertising control) were shown a video, produced by an overseas primary school, providing five child backpack safety guidelines for parents (i.e. check straps and backpack size, adjust backpack, wear it right, pack smart, brighter is better). This video was edited to be of similar length to the ‘Our kids are sweet enough’ video (1 min 52 s) and to remove any references not relevant to an Australian audience.

### Measures

#### Purchasing preferences

To assess advertising impacts on generic and branded purchasing preferences respectively, parents were asked to imagine they were shopping tomorrow and buying snacks for their child. In two separate tasks, they were shown (i) five generic products (fruit-flavoured confectionery, fruit straps or fruit leathers, fruit puree, dried fruit, whole fruit) and (ii) six branded products and prompted to select from the set of products which they would be most likely to buy. The six products featured in the branded product preference task were the advertised conventional confectionery brand and a non-advertised conventional confectionery brand, the advertised pseudo-healthy confectionery brand and a non-advertised pseudo-healthy confectionery brand, and two brands of whole fruit kids packs. The two non-advertised confectionery brands were chosen based on their similarity to the corresponding advertised brand as well as their appeal to children. Separate binary variables were created to indicate whether parents selected the branded product advertised in the conventional or pseudo-healthy confectionery ads, or if they chose a whole fruit product in line with the message of the counter-ad. Additional binary variables denoting whether parents selected each option in the generic purchasing preference task were also created.

#### Brand attitudes

Parents’ attitude towards the conventional and pseudo-healthy confectionery food brands promoted in the product advertising were measured using a 7-point semantic differential scale anchored by negative/positive.

#### Product perceptions

Using 7-point scales, parents rated each of the generic and branded products displayed in the purchasing preference task in terms of their level of healthiness (1 = ‘not healthy at all’ to 7 = ‘very healthy’), sugar content (1 = ‘no sugar’ to 7 = ‘high sugar’) and fibre content (1 = ‘no fibre’ to 7 = ‘high fibre’).

#### Policy support

To gauge parents’ level of support for food policies related to the counter-advertising, parents were asked to indicate whether they would be in favour or against government taking the following actions to support healthy eating: “Introducing regulations so that food companies can’t promote healthy aspects of foods that are mostly unhealthy”; “Making it compulsory for all packaged foods to display a Health Star Rating on the front of pack”; “Requiring the amount of added sugar to be separated out from naturally occurring sugars in the nutrition information panel”. Responses were recorded on a 7-point scale ranging from 1 = ‘strongly against’ to 7 = ‘strongly in favour’.

### Statistical analysis

Data were analysed using Stata/MP V.16.1 [59]. A combination of logistic (for binary outcomes) and linear (for continuous outcomes) regression models were run to test for differences by advertising condition on brand purchasing preferences, attitudes and product perceptions, with the non-food advertising (control) condition specified as the reference category. Planned comparisons (condition B vs. D; condition C vs. E) were conducted to assess the effects of exposure to the counter-advertising relative to exposure to conventional or pseudo-healthy confectionery advertising only. For the generic purchasing preferences and product perceptions and policy support outcomes, initial models (excluding the control condition) were run with an interaction term between counter-advertising (unexposed/exposed) and type of confectionery advertising (conventional/pseudo-healthy). As only one of the 23 interactions tested was statistically significant, these interaction terms were omitted from the final models; however, type of confectionery advertising was retained as a covariate.

## Results

### Purchasing preferences

**Fig. 1:**
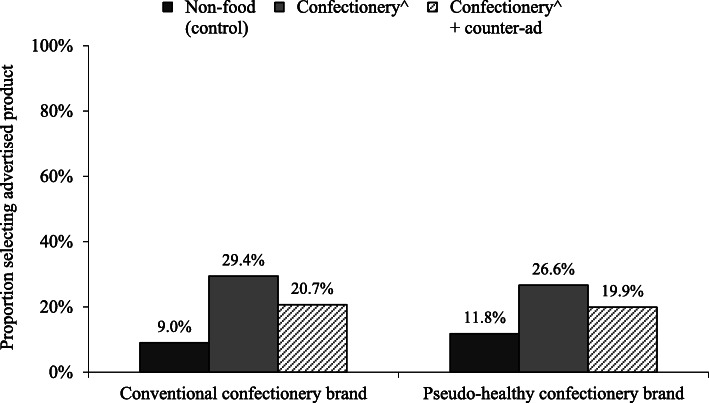
Proportion of parents indicating a preference to purchase the advertised product by advertising condition. ^ Conventional confectionery for proportion selecting ‘Conventional confectionery brand’ and Pseudo-healthy confectionery for proportion selecting ‘Pseudo-healthy confectionery brand’

As Fig. 1 illustrates, consistent with expectations (H1a), parents in the two confectionery advertising only conditions were more likely to choose their advertised product in the branded purchasing preference task compared to parents in the control condition (conventional: OR = 4.21, 95% CI: 2.68–6.60, *P* < 0.001; pseudo-healthy: OR = 2.71, 95% CI: 1.78–4.12, *P* < 0.001). Further, as hypothesised (H2a), parents who were exposed to the counter-advertising intervention in addition to either type of confectionery advertising were less likely to choose their advertised product than those who were not exposed to the counter-advertising intervention (conventional: OR = 0.63, 95% CI: 0.44–0.90, *P* = 0.011); pseudo-healthy: OR = 0.69, 95% CI: 0.47–0.99, *P* = 0.045). However, it should be noted that parents who viewed the counter-advertising were still more likely to choose their advertised product when compared to parents in the control condition, who saw no confectionery advertising at all (conventional: OR = 2.63, 95% CI: 1.65–4.20, *P* < 0.001; pseudo-healthy: OR = 1.86, 95% CI: 1.20–2.88, *P* = 0.005).

In line with H2b, exposure to the counter-advertising prompted a higher proportion of parents to select a whole fruit snack option in the branded purchasing preference task compared to exposure to either type of confectionery advertising only (conventional: 60.2% vs. 50.8%, OR = 1.47, 95% CI: 1.07–2.00, *P* = 0.016; pseudo-healthy: 60.7% vs. 48.9%, OR = 1.62, 95% CI: 1.18–2.21, *P* = 0.003). However, no effects of the counter-advertising were found for the generic purchasing preference task (all *P* > 0.05).

### Brand attitudes

As hypothesised (H1b), parents who viewed conventional confectionery advertising held more positive attitudes towards the advertised brand than parents in the control condition (M = 4.64 vs. M = 4.38; β = 0.06, *P* = 0.048). However, contrary to H1b, viewing pseudo-healthy confectionery advertising did not influence parents’ brand attitudes compared to viewing control advertising (M = 5.30 vs. M = 5.23; β = 0.02, *P* = 0.520).

In line with expectations (H2a), parents’ attitudes towards their advertised brand were less positive if they had been exposed to the counter-advertising compared to if they had been unexposed (conventional: M = 4.33 vs. M = 4.64; β = − 0.07, *P* = 0.017; pseudo-healthy: M = 5.01 vs. M = 5.30; β = − 0.08, *P* = 0.009).

### Product perceptions

In line with H1c, parents exposed to pseudo-healthy confectionery advertising perceived the advertised product to be significantly healthier and contain less sugar and more fibre compared to parents in the control condition (see Table 2). Unexpectedly, parents exposed to conventional confectionery advertising also perceived the advertised product to be healthier and contain less sugar than parents in the control condition, while no such effect was found for perceptions of fibre content.

**Table 2:** Parent perceptions of the health and nutritional attributes of the advertised product by advertising condition

	Advertising condition					
	Non-food (control)		Confectionery^c^		Confectionery^c^ + counter-ad	
	*M*	*SD*	*M*	*SD*	*M*	*SD*
**Healthiness**						
Conventional confectionery brand	2.48	1.77	2.76^a*^	1.87	2.59	1.91
Pseudo-healthy brand	3.09	1.75	3.80^a***^	1.73	2.88^b***^	1.75
**Sugar content**						
Conventional confectionery brand	6.01	1.40	5.63^a**^	1.55	5.95^b**^	1.46
Pseudo-healthy brand	5.54	1.51	5.06^a***^	1.50	5.48^b***^	1.60
**Fibre content**						
Conventional confectionery brand	2.40	1.81	2.67	1.85	2.31^b*^	1.76
Pseudo-healthy brand	2.93	1.79	3.38^a**^	1.73	2.72^b***^	1.73

^*^P < 0.05; ^**^ *P* < 0.01; ^***^ *P* < 0.001

^a^Significant difference compared to non-food (control) advertising condition

^b^Significant difference compared to confectionery advertising condition

^c^Branded advertising for the respective conventional and pseudo-healthy confectionery products

As predicted (H2a), parents perceived the advertised product to have more sugar and less fibre if they had been exposed to the counter-advertising compared to if they had viewed the confectionery advertising only. Parents who viewed the pseudo-healthy confectionery advertising also rated the advertised product as less healthy if they had been exposed to the counter-advertising; a similar effect of the counter-advertising on this perception was not found for those who saw the conventional confectionery advertising (see Table 2).

Across conditions, parents generally perceived whole foods (i.e. whole or dried fruit) to be healthier and contain lower sugar and higher fibre than processed foods (i.e. fruit-flavoured confectionery, fruit straps or fruit leathers, fruit puree). Consistent with H2a, parents’ ratings of the healthiness and nutrition content of the processed foods were less favourable following exposure to the counter-advertising (see Table 3). The only exception was in relation to perceptions of the sugar content of fruit-flavoured confectionery, with parents rating this generic product similarly high in sugar regardless of whether they had seen the counter-advertising. Exposure to the counter-advertising also prompted parents to perceive dried fruit less favourably in terms of healthiness and fibre content whereas perceptions of the sugar and fibre content of whole fruit became more favourable as a result of viewing the counter-advertising.

**Table 3:** Parent perceptions of the health and nutritional attributes of generic products by exposure to counter-advertising

	Counter-advertising			
	Unexposed^a^		Exposed^b^	
	*M*	*SD*	*M*	*SD*
**Healthiness**				
Fruit-flavoured confectionery	2.76	1.81	2.49^**^	1.79
Fruit straps/leathers	3.57	1.62	2.90^***^	1.71
Fruit puree	4.34	1.43	3.68^***^	1.66
Dried fruit	5.22	1.29	4.87^***^	1.44
Whole fruit	6.47	0.98	6.50	1.02
**Sugar content**				
Fruit-flavoured confectionery	6.01	1.29	6.06	1.42
Fruit straps/leathers	5.42	1.34	5.73^***^	1.40
Fruit puree	5.07	1.28	5.28^**^	1.39
Dried fruit	4.56	1.57	4.43	1.61
Whole fruit	4.00	1.69	3.62^***^	1.61
**Fibre content**				
Fruit-flavoured confectionery	2.48	1.78	2.19^**^	1.68
Fruit straps/leathers	3.34	1.71	2.65^***^	1.66
Fruit puree	4.01	1.57	3.40^***^	1.71
Dried fruit	5.14	1.41	4.84^***^	1.58
Whole fruit	5.92	1.23	6.10^**^	1.30

^**^ *P* < 0.01; ^***^ *P* < 0.001 denotes significant difference compared to unexposed counter-advertising conditions (i.e. B and C)

^a^Includes Conventional confectionery and Pseudo-healthy confectionery advertising conditions (i.e. B and C)

^b^Includes Conventional confectionery + counter-advertisement and Pseudo-healthy confectionery + counter-advertisement conditions (i.e. D and E)

### Policy support

There was some evidence that the counter-ad increased support for policy concerning food labelling and marketing (RQ1). Specifically, compared to parents who viewed confectionery advertising only, those who were also exposed to counter-advertising showed higher support for introducing regulations prohibiting food companies from promoting healthy aspects of foods that are mostly unhealthy (M = 5.87 vs. M = 5.61; β = 0.10, *P* = 0.001) and requiring added sugar to be listed separately in the nutrition information panel (M = 5.83 vs. M = 5.64; β = 0.07, *P* = 0.014). Support levels for making it compulsory for all packaged foods to display a Health Star Rating on the front of pack were unaffected by the counter-advertising.

## Discussion

Our results indicate both the conventional and pseudo-healthy confectionery **product ads** achieved outcomes typically sought by food advertisers, namely, boosting preference for and perceptions of the advertised products. Despite employing some different persuasive appeals, both types of ad more than doubled the odds of parents choosing the advertised product in the preference task and significantly enhanced nutrition-related perceptions of the advertised product, effectively conferring a ‘health halo’ on the advertised product. The conventional confectionery ad employing predominantly pleasure-based sensory appeals was especially impactful on product preferences and enhancing overall brand attitudes, while the pseudo-healthy confectionery ad was especially impactful in enhancing nutrition-related product perceptions reflective of the persuasive tactics used in that ad. It is notable that the conventional confectionery ad also enhanced some nutrition and health-related perceptions of advertised products, even though this was not a theme of the ad. As nutritional concerns are just one factor that drives food preferences, ads that tap into other important preference drivers, such as anticipated taste and pleasure, may be particularly influential in boosting brand preference and overall brand image [60]. While the ads we tested promoted child-oriented products, it is notable that the persuasive appeals they employed (fun, flavour, family life and, in the case of the pseudo-healthy confectionery product, nutrition) appear to have influenced our sample of parents, highlighting that impacts of unhealthy food advertising are not exclusive to children.

In contrast to the product ads, the **counter-ad** diminished preferences for the advertised brand and increased preference for whole fruit products. The counter-ad also detracted from brand attitudes promoted by the product ads and helped correct parents’ perceptions concerning the advertised products’ sugar and fibre content and overall level of healthiness. The counter-ad also increased parents’ support for regulations prohibiting food companies from promoting healthy aspects of otherwise unhealthy products and requiring added sugar to be listed separately on the nutrition information panel. Our findings among a sample of parents exposed to a video-based counter-ad that presented inoculation-style messaging in a narrative format echo those of Mason et al., who found text-based inoculation messages offered an effective strategy for helping to protect young adults’ health conscious attitudes and made them resistant to potentially deceptive nutrition-related advertising claims [43]. They also correspond with earlier research suggesting counter-ads can help bolster parents’ resistance to influence by common front-of-pack promotions used to promote unhealthy foods [37]. Further, these findings are consistent with meta-analyses which found narrative health communications can be persuasive, especially when delivered in an audio-visual format [53] and inoculation messages can be an effective strategy to induce resistance to persuasion [42]. Together these results are encouraging, in that they suggest that health communication interventions designed to expose and contest potentially misleading advertising tactics can deliver measurable impacts on consumers’ attitudes and preferences, which are more aligned with public health nutrition recommendations and advocacy. Findings also point to a potential role for parents who have concerns about adverse impacts of unhealthy food marketing to advocate for higher standards for how child-oriented food products can be marketed. Some advocacy-oriented parent groups are already active in this space, such as ‘Parents’ Voice’, an online network of parents who are interested in improving the food and activity environments of Australian children [61].

### Implications

While the findings for the counter-ad were encouraging, it is notable that the scenario that promoted least preferences and favourable attitudes toward the unhealthy brands was when parents were exposed to no product advertising at all (control condition). This suggests regulations prohibiting unhealthy food product advertising would offer the most effective route for reducing demand for unhealthy products. This finding lends support to the approach taken in a growing number of jurisdictions (currently 18 countries) where certain forms of food advertising or commercial promotion (especially those targeted at children) are restricted or banned [30]. This contrasts with Australia, where despite high rates of overweight and obesity among children and adults, there are few controls on advertising practices targeting ads for unhealthy foods and beverages to children and a reliance on self-regulation by the food and beverage industry [62]. In such settings where statutory restrictions on unhealthy food advertising and marketing have not been implemented, counter-advertising strategies offer a promising intervention alternative that may go some way towards detracting from persuasive impacts of unhealthy food advertising and complement advocacy efforts for improved regulatory controls to reduce the public’s exposure to such marketing and advertising. Nonetheless, in the absence of advertising restrictions, it is an ambitious task for public health counter-advertising campaigns to achieve a sufficiently strong reach and frequency of audience exposure to effectively counter the persuasive power of product advertising campaigns afforded by the food industry.

### Limitations

There are limitations to the present online experimental study. Firstly, because we tested parents’ reactions to actual food product ads that are typical of those repeatedly aired on mainstream media, it is likely participants had previously seen many similar ads, such that the single product advertising exposure they received in our study could have been supplemented by many previous exposures. In contrast, the counter-ad we tested had only previously been accessible to the public via a weblink on the Obesity Policy Coalition’s website or YouTube, such that participants were unlikely to have previously been incidentally exposed to this intervention. Therefore, participants are likely to have had a higher total ‘dose’ of product advertising relative to counter-advertising at the time of this study. Although this research indicates that exposing parents to a single ‘dose’ of counter-advertising helped diminish persuasive effects of subsequently viewed food advertising, it is possible that demand effects may have led parents to respond to the counter-advertising (and product advertising) as intended given that exposure was forced. While a short distractor task was undertaken between seeing the advertisements and completing the response measures, no masking questions were included. Further, because our behavioural measure was a simulated food preference task, we cannot be certain how the product advertising and counter-advertising would impact actual purchasing and consumption, given that multiple other factors such as price and physical positioning are also influential factors not considered in the current study [63, 64]. Future research testing longer-term impacts of counter-advertising on actual behaviour would extend this area of enquiry.

Because online non-probability samples do not provide a random population sample, it is not possible to generalise our results to the national population of parents. Nonetheless, online panels are useful for predicting consumer responses to advertising and providing randomisation to different exposure conditions. Our sample comprised roughly even proportions of males and females and a good spread in terms of socio-economic position, so the pattern of responses we observed in this large, diverse sample of parents may reflect those in the population.

This was an opportunistic study where we tested an existing counter-ad. While this counter-ad contains certain elements indicative of inoculation messaging, we acknowledge it is not a pure exemplar of this theoretically based message format, as it was not originally produced with that intention. Nonetheless, our findings echo those of another study that did use theoretically-based inoculation messages to confer resistance to potentially deceptive health nutrition-related advertising claims [43]. Findings also converge with our earlier counter-advertising research with parents [37]. Further research exploring the impacts of various approaches to theoretically informed, narrative-style counter-advertising is warranted. It would also be of interest to systematically test whether exposing consumers to some of the food related ‘subvertising’ generated through the anti-consumerist movement affords consumers any protection against influence by advertising for unhealthy food and drink products.

Despite some limitations, this study adds to the evidence base in several ways. Firstly, it helps extend the focus of research examining impacts of unhealthy food advertising to parents, who are an important target group for food advertising because of their influential role in purchasing and providing food to their family. It also tested reactions to different types of persuasive appeals commonly used to advertise unhealthy food products, including the increasingly common use of health and nutrition-related appeals. The study also moves beyond simply documenting problematic impacts of unhealthy food advertising to exploring the utility of counter-advertising in helping to remedy these impacts. By using an existing professionally produced, video-based counter-ad, we were able to extend previous research which tested text-only [43] or banner-ad only unhealthy food counter-advertising interventions [37].

## Conclusions

By testing responses to typical, ‘real-world’ food product advertising and to counter-advertising that could feasibly be disseminated through mass media, this study provides practical evidence that could readily be translated into policy and practice to assist with population-level obesity prevention efforts. Even brief exposure to unhealthy product advertising influenced parents’ product preferences and perceptions. Encouragingly, results also demonstrated that counter-advertising holds promise in empowering parents to more accurately evaluate advertised food products. Improving our understanding of the impacts of food marketing on consumers and testing the efficacy of interventions to diminish this influence should help to identify and implement evidence-based corrective policies and practices. In the absence of restrictions on unhealthy food advertising, counter-ads may go some way towards detracting from persuasive effects of unhealthy food advertising.

## Supplementary Information

**Additional file 1.** Counter-advertisement voice-over script and still image. This file contains the voice-over script of the *Our Kids Are Sweet Enough* video produced by the Obesity Policy Coalition in Australia.

## Data Availability

The data used and analysed in the current study are available from the corresponding author on reasonable request.
