# Supplementary material for: Can counter-advertising diminish persuasive effects of conventional and pseudo-healthy unhealthy food product advertising on parents?: an experimental study
Source: BMC Public Health. 2020 Nov 25;20:1781. doi: 10.1186/s12889-020-09881-1 (PMC7687848; doi:10.1186/s12889-020-09881-1)
Supplement: Supplementary file 1 — Additional file 1. Counter-advertisement voice-over script and still image. This file contains the voice-over script of the Our Kids Are Sweet Enough video produced by the Obesity Policy Coalition in Australia. [file 12889_2020_9881_MOESM1_ESM.pdf]

Additional file 1: Counter-advertisement voice-over script and still image

Our Kids Are Sweet Enough – Obesity Policy Coalition

Once upon a time, there was a delicious, healthy apple called Alfie. Alfie was full of fibre, vitamins and minerals – lots of things our bodies need. But this piece of fruit wasn't destined to end up sitting merrily in someone's lunchbox. No, no. This poor piece of fruit was headed to a factory. Here, food manufacturers would remove nearly all his goodness by sieving him, boiling him and removing all his water until he was barely more than a pile of sugar. Then, they pushed him through a metal extruder and dried him out on a production line. This sticky paste no longer resembled the nutritious piece of fruit Alfie the apple once was. The paste was incredibly high in sugar and low in natural fibre. Alfie was no longer good for our bodies, but instead, bad for our teeth and waist-lines. What's worse, this sugary paste would be used in so-called 'healthy' products for adults, kids and even toddlers. Food marketers will use him as a promotional tool; a way to sell their unhealthy products to Mums and Dads wanting to give their kids a healthy life. Don't be tricked – leave Alfie as he is, along with his healthy, delicious, whole-fruit and veg mates. Our kids are sweet enough.

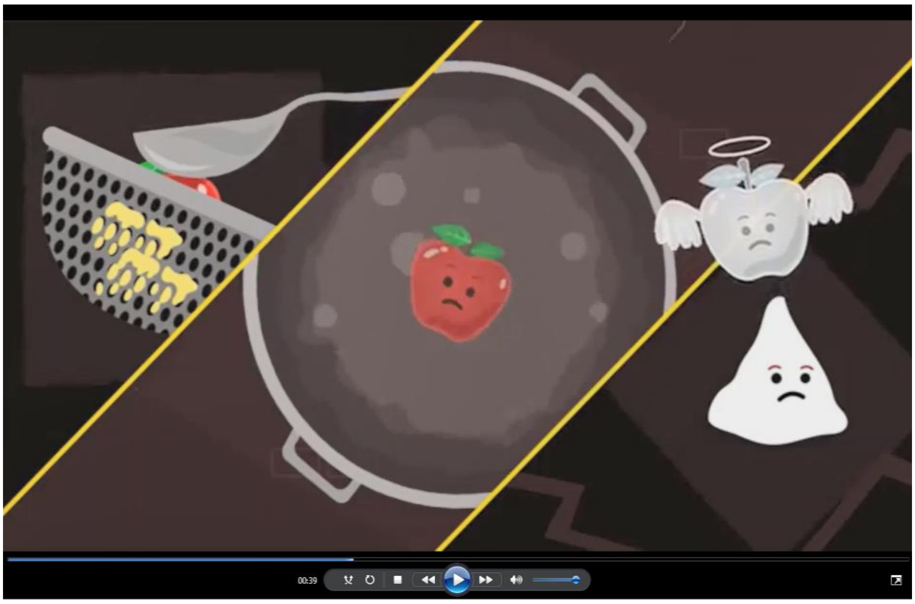

Link to video: https://www.opc.org.au/what-we-do/kids-are-sweet-enough
